# Investigation and analysis of rehabilitation therapists' current situation in Hunan province after the COVID-19 epidemic: a cross-sectional study

**DOI:** 10.3389/fresc.2025.1614160

**Published:** 2025-11-20

**Authors:** Jun Wang, Jiachun Yang, Zhiyong Duan, Yue Huang, Na Deng

**Affiliations:** 1 Department of Rehabilitation Medicine, Pingshan District Central Hospital of Shenzhen, Shenzhen, China; 2 Department of Rehabilitation Medicine, The Second Xiangya Hospital of Central South University, Changsha, Hunan, China; 3 Department of Rehabilitation Medicine, Affiliated Hospital of Guangdong Medical University, Guangdong, China; 4 Department of Rehabilitation Medicine, Taojiang County Nursing and Rehabilitation Hospital, Yiyang, Hunan, China; 5 Department of Adult Rehabilitation, Xiangya Boai Rehabilitation Hospital, Changsha, Hunan, China

**Keywords:** coronavirus, epidemic, job satisfaction, rehabilitation therapists, COVID-19

## Abstract

**Background:**

This study examines the current status of rehabilitation therapists in Hunan Province after the COVID-19 outbreak and identifies factors affecting their job satisfaction.

**Objective:**

The purpose of this article is to understand the post-pandemic conditions of rehabilitation therapists in Hunan Province.

**Method:**

After expert review, a cross-sectional questionnaire was disseminated via WeChat using the Wenjuanxing platform. Of 809 questionnaires collected, 769 valid responses were included in the final analysis after excluding incomplete or inconsistent data.

**Results:**

This study analyzed the relationships between demographic factors and job satisfaction among rehabilitation therapists. Significant differences were found in age, work experience, job title, and monthly salary level (*P* < 0.001). Notably, lower income was strongly associated with greater job dissatisfaction; each one-category increase in monthly income was associated with 51% lower odds of being in a higher (more dissatisfied) category of job satisfaction (OR = 0.49, 95% CI: 0.42–0.57; *P* < 0.001), underscoring income as a key predictor of job satisfaction.

**Conclusions:**

Our investigation reveals that rehabilitation therapists' roles in Hunan have become more specialized, with monthly income emerging as a critical driver of job satisfaction. These findings underscore the need for competitive compensation and wider professional support to maintain workforce stability. By focusing on the post-pandemic landscape in Hunan Province, this study offers valuable insights that may guide policy and practice for rehabilitation therapists across China.

## 1 Introduction

The significant COVID-19 pandemic, which began in 2019, rapidly spread worldwide and caused major changes in human behavior and economic patterns (1). Changes in human behavior can largely be attributed to the novel coronavirus's transmission pathways. Infection occurs primarily through close contact with an infected individual who has coughed, sneezed, or released aerosols into the environment. These aerosols can enter the human respiratory system through inhalation via the the nose or mouth (2, 3). This has led people to maintain a degree of social distance during interpersonal interactions.

According to the World Health Organization (WHO) update on August 17, 2023, the COVID-19 pandemic has spread to over 200 countries. So far, approximately 6,955,141 people have died from COVID-19 out of nearly 769,774,646 confirmed cases (4). Given the high incidence and mortality rates, many economies worldwide have experienced varying degrees of contraction or stagnation. A significant number of individuals lost their jobs and struggled to meet their families' financial needs (5, 6).

Deteriorating economic conditions, reduced patient admissions, and widespread COVID-19 transmission have significantly impacted the healthcare sector. The elements and emphasis of rehabilitation therapies within healthcare institutions have undergone significant transformations. Prior to the emergence of the COVID-19 pandemic, the routine clinical responsibilities of rehabilitation therapists were diverse and comprehensive (7, 8). With the emergence of the pandemic, focused primarily on respiratory and critical care rehabilitation. Not only can the COVID-19 virus affect the pulmonary system (9), but it also increases the mortality rate (10). The findings of the study conducted by Veronical et al. indicate that early-stage mobilization and physical exercise are advantageous for individuals diagnosed with COVID-19 (11). Moreover, pulmonary rehabilitation has also been shown to be beneficial for COVID-19 patients (12, 13). Rehabilitation now places greater focus on pulmonary recovery and intensive care methods. This shift has posed considerable challenges, diverging markedly from traditional vocational trajectories. The situation required advanced competencies in pulmonary rehabilitation and urgent recovery strategies. Simultaneously, there has been a shift in patient behaviors. Individuals considering hospital visits have delayed their medical appointments due to an increased vulnerability to coronavirus infections and a lack of available medical resources (14, 15). The current shortage of resources is largely attributable to the pressing needs associated with the care of patients affected by COVID-19. As a result, there is a significant risk that the financial situation of rehabilitation therapists may deteriorate, stemming from a reduced patient volume and an ensuing decrease in their earnings.

On January 8th, 2023, China lifted its epidemic control measures (16). This transition is clearly reflected in a significant increase in hospital visits for medical treatment. Compared with the pandemic period, our observations show a distinct rise in the number of patients seeking medical care. This increase is further correlated with a rise in income for rehabilitation therapists. By applying COVID-19 guidelines and leveraging relevant expertise, rehabilitation therapists may reduce their workload in caring for COVID-19 patients.

Despite extensive database searches, limited research exists on rehabilitation therapists' post-COVID-19 status. Grounded in the Job Demands–Resources framework and effort–reward imbalance theory, we conceptualize income and job title as core resources/rewards that should positively relate to job satisfaction, whereas longer tenure without advancement may signal unmet reward expectations. Accordingly, we hypothesized that higher income and more senior titles would be associated with higher odds of reporting greater job satisfaction, adjusting for demographic and institutional factors. Given potential contextual heterogeneity across institutions, we also explored whether the strength of these associations varied by work unit. So, this study examines their professional conditions in Hunan, focusing on job satisfaction determinants such as income and work experience. Using a cross-sectional quantitative survey, it aims to provide valuable insights into the rehabilitation therapist community, offering a foundation for understanding their nationwide challenges.

## 2 Method

### 2.1 Study design and participants

This cross-sectional survey was conducted in Hunan Province between June and July 2023. Ethical approval was obtained from the Ethics Committee of Xiangya Boai Rehabilitation Hospital prior to data collection. Hunan was among the first regions in China to implement university-based rehabilitation therapy programs [in 1997 (17)] and has a substantial rehabilitation workforce and diverse institutional settings. These features provide contextual breadth and heterogeneity; however, the sample should not be considered nationally representative. The target population comprised rehabilitation therapists employed in hospitals or rehabilitation clinics in Hunan, including physical therapists, occupational therapists, speech therapists, cardiopulmonary therapists, postpartum rehabilitation therapists, pediatric therapists, and prosthetists and orthotists. Inclusion criteria were current employment as a rehabilitation therapist in Hunan. We imposed no restrictions on gender, age, unit ownership (public/private), facility level/type (tertiary/secondary/primary), rehabilitation specialty, geographic location within Hunan, academic major, job title, years of experience, monthly income, or degree.

A convenience sample was recruited via the internet. To efficiently reach this professional population in the post-COVID period, we adopted a two-stage approach: (1) at the organizational-network level, the research team collaborated with the provincial rehabilitation medicine association and hospital departments to disseminate the survey announcement and Wenjuanxing link across multiple professional WeChat groups (association channels, hospital department groups, alumni networks) representing different hospital levels (tertiary/secondary/primary), geographic areas (urban/suburban/rural), and specialties; and (2) at the individual level, convenience sampling was used within these groups. Therapists who saw the announcement could voluntarily access the link, provide electronic consent, and complete the questionnaire. We maintained a dissemination log tracking group categories, hospital levels, and regions reached. The survey window remained open for two months to maximize reach and heterogeneity. Group administrators were asked to avoid targeted invitations to specific subgroups to minimize selection pressure.

Given online convenience sampling through professional WeChat groups, several selection mechanisms may operate: network participation bias (therapists active in professional groups may be overrepresented, whereas those with limited internet access or not engaged in WeChat may be underrepresented); self-selection bias (individuals with stronger opinions about job satisfaction may be more likely to respond); and institutional clustering (respondents nested within institutions may share policies and cultures, inducing intra-cluster correlation). To mitigate these limitations, we took the following steps: broad dissemination across hospital levels, regions, and specialties; a prolonged survey window to capture different schedules; and pre-specified inclusion/exclusion criteria with duplicate/inconsistency checks. Nevertheless, the sample should be interpreted as a non-probability online sample of Hunan rehabilitation therapists. Findings may not generalize to therapists who are offline, not active in professional WeChat networks, or working outside Hunan. Future studies should consider probability-based or stratified sampling across provinces and multi-mode recruitment (online/offline) to enhance external validity.

This study identifies job satisfaction as the primary outcome. Job satisfaction, characterized as a positive or negative emotional response related to one's current work conditions, serves as a meaningful indicator of the experiences of rehabilitation therapists in the aftermath of the pandemic. Furthermore, employing ordinal logistic regression allows for a systematic examination of the factors that influence job satisfaction.

### 2.2 Procedure

Building on a questionnaire framework informed by literature review (18) and expert discussions at the hospital, we developed the final survey on the Wenjuanxing platform. To ensure content validity and clarity, the instrument underwent multiple expert revisions. We then conducted cognitive testing and a pilot with ten rehabilitation therapists; feedback was used to refine or remove items that were illogical or ambiguous.

The finalized survey link was distributed as described in Section 2.1. The survey was administered anonymously, and all participants provided electronic informed consent before proceeding. Two reviewers independently screened submitted questionnaires for data integrity and internal consistency (e.g., mismatches between years of service and professional titles); disagreements were adjudicated by a third reviewer. Following these quality checks, completed and valid questionnaires were locked for analysis at the end of the pre-specified two-month data collection window (Supplementary Material S1).

### 2.3 Demographic data

The survey consists of three sections: personal details, professional background, and career expectations. Personal details include gender, age, kind of work unit, type of work unit, area of rehabilitation work, workplace location, major, and degree. Professional background section includes job title, years of work experience, and monthly income level. Finally, the career expectations section includes a job satisfaction scale consisting of single items rated on a 5-point Likert scale (1 = very satisfied, 5 = very dissatisfied). These items measure various aspects of job satisfaction, including work environment, salary, and professional background.

### 2.4 Data analysis

Data from Wenjuanxing were first exported to Excel 2019 (Microsoft Corporation, USA) for descriptive analysis. Following this, we used the statistical analysis methods in Excel 2019 to perform a detailed analysis of the collected data. We also calculated data frequency, composition ratios, and other relevant statistics.

IBM SPSS Statistics version 24 was used for statistical analyses. In the analysis of demographic data, categorical variables—including gender, type of work unit, kind of work unit, work location, work content, job title, major, degree, job satisfaction, and monthly income level—were examined using SPSS statistical software to compute their composition ratios. For the quantitative variables of age and years of service, SPSS was employed to assess whether these variables adhered to a normal distribution. If the data were normally distributed, they were represented as mean ± standard deviation, and one-way ANOVA was used for statistical evaluation. Conversely, if they were not normally distributed, median and interquartile range were used, followed by the Kruskal–Wallis test. Construct validity of the single-item job satisfaction scale was assessed through convergent and known-groups methodologies following established measurement standards. Nonparametric statistical techniques were applied to examine associations and group differences. To identify and control for potential confounding variables, such as personal details and professional background, we included these factors in ordinal logistic regression. By adjusting for these confounders, we aimed to ensure that any significant associations observed were reflective of true relationships rather than external biases. We report regression coefficients (B), standard errors (S.E.), odds ratios (OR), 95% confidence intervals (CI), and *p*-values. Model fit and parsimony were assessed using Akaike's Information Criterion (AIC), Bayesian Information Criterion (BIC), and the goodness-of-fit measure McFadden pseudo-R^2^. Variance inflation factors (VIF) were inspected to gauge multicollinearity. As a sensitivity analysis, we re-estimated the model with robust standard errors clustered at the work-unit level; inferences were materially unchanged. The significance level was set at 0.05.

## 3 Result

Regarding the questionnaire's job satisfaction assessment, participants answered a series of questions related to work environment, salary, and professional background, and overall satisfaction using a 5-point Likert scale. These responses allowed us to quantitatively analyze factors influencing job satisfaction, thus connecting demographic data with satisfaction outcomes.

In this study, a total of 809 samples were collected and rigorously screened for validity. After excluding 40 (4.9%) invalid samples due to inconsistencies in recorded years of experience (10 cases, 1.2%) and incomplete information on key variables such as monthly salary and job title (30 cases, 3.7%), the final sample size consisted of 769 (95%) valid cases, ensuring the integrity of the analysis.

Among the participants, the majority were female (61.8%), and 52.4% were under the age of 30. Most rehabilitation therapists practiced in public hospitals (78.3%), had less than 10 years of professional experience (81.9%), and held technologist-in-charge positions (41.2%). Education levels were concentrated at the bachelor's degree level (68.7%), and the majority reported a monthly income of 5,000 yuan or below. For a detailed breakdown of demographic characteristics, please refer to Table 1.

**Table 1:** Demographic characteristics of participants.

Variables	Categories	*n*	*n*/*N* (%) Percentage
Gender	Male	294	38.2%
	Female	475	61.8%
Age	<30 years	403	52.4%
	30–40 years	277	36.0%
	>40 years	89	11.6%
Kind of work unit	Public hospital	602	78.3%
	Private hospital	167	21.7%
Type of work unit	General hospital	591	76.9%
	Rehabilitation hospital	127	16.5%
	Rehabilitation clinic	43	5.6%
	Special hospital	8	1.0%
Workplace location	Capital city	289	37.6%
	Non-capital city	480	62.4%
Work content	Physical therapist	476	61.9%
	Occupational therapist	87	11.3%
	Speech therapist	101	13.1%
	Cardiopulmonary therapist	33	4.3%
	Postpartum rehabilitation therapist	9	1.2%
	Pediatric therapist	22	2.9%
	Prosthetic orthotist	12	1.6%
	Concurrent post	29	3.8%
Years of work experience	<5 years	309	40.2%
	5–10 years	321	41.7%
	>10 years	139	18.1%
Job title	Technician	73	9.5%
	Technologist	299	38.9%
	Technologist-in-charge	317	41.2%
	Associate senior technologist	69	9.0%
	Senior Technologist	11	1.4%
Major	Rehabilitation therapy	517	67.2%
	Physical therapy	22	2.9%
	Speech therapy	14	1.8%
	Occupational therapy	17	2.2%
	Acupuncture and manipulation	119	15.5%
	Clinical medicine speciality	46	6.0%
	Nursing	34	4.4%
Degree	Secondary school diploma	9	1.2%
	Associate degree	170	22.1%
	Bachelor degree	528	68.7%
	Master degree	56	7.3%
	Doctor degree	6	0.8%
Monthly salary level	<3,000 RMB	86	11.2%
	3,000–5,000 RMB	328	42.7%
	5,001–7,000 RMB	206	26.8%
	7,001–10,000 RMB	94	12.2%
	>10,000 RMB	55	7.2%
Job satisfaction	Very satisfied	49	6.4%
	Satisfied	245	31.9%
	Average	371	48.2%
	Dissatisfied	62	8.1%
	Very dissatisfied	42	5.5%

Percentages are based on non-missing responses; denominators equal *N* = 769 for all variables in the final analytic sample unless otherwise specified.

Percentages may not sum to 100% due to rounding; Job title is ordered as Technician < Technologist < Technologist-in-charge < Associate senior technologist < Senior technologist; Monthly income categories: <3,000; 3,000–5,000; 5,001–7,000; 7,001–10,000; >10,000 RMB.

The single-item exhibited no pronounced ceiling/floor effects (5.5% and 6.4%, respectively). In convergent validity analyses, job satisfaction correlated significantly with monthly income level (*ρ* = −0.39, *P* < 0.001), years of experience (*ρ* = −0.20, *P* < 0.001), and job title (*ρ* = −0.20, *P* < 0.001), and showed a borderline association with educational degree (*ρ* = −0.09, *P* = 0.010). The negative signs reflect coding polarity and remain consistent with the hypothesized directions after recoding (higher income and seniority correspond to higher satisfaction). Known-groups validity was supported by significant differences across job title groups (X^2^ = 36.6, *P* < 0.001). Internal consistency was not applicable given the single-item nature (Supplementary Material S2).

Statistical analysis revealed significant differences in age (H = 32.90, *P* < 0.001), years of work experience (H = 32.14, *P* < 0.001), job title (H = 29.93, *P* < 0.001), and monthly salary level (H = 114.81, *P* < 0.001) with respect to job satisfaction. However, no statistically significant differences were observed for gender (H = 4.15, *P* = 0.38), kind of work unit (H = 8.86, *P* = 0.06), type of work unit (H = 5.97, *P* = 0.201), workplace location (H = 5.00, *P* = 0.28), or degree (H = 9.35, *P* = 0.053). For more details, see Table 2.

**Table 2:** Bivariate associations with job satisfaction.

Variable	Total (*n* = 769)	Very satisfied (*n* = 49)	Satisfied (*n* = 245)	Average (*n* = 371)	Dissatisfied (*n* = 62)	Very dissatisfied (*n* = 42)	Statistic	*P*
Age M (Q₁, Q₃)	29.00 (25.00–35.00)	32.00 (28.00–39.00)	30.00 (26.00–37.00)	29.00 (25.00–33.00)	29.00 (24.00–32.75)	26.00 (22.25–28.75)	H = 32.90	<0.001
Years of work experience M (Q₁, Q₃)	6.00 (2.00–10.00)	9.00 (4.00–13.00)	7.00 (3.00–11.00)	5.00 (2.00–9.00)	5.00 (2.00–7.75)	3.00 (1.00–6.75)	H = 32.14	<0.001
Gender, *n* (%)							H = 4.15	0.38
Male	294 (38.23)	23 (46.94)	97 (39.59)	134 (36.12)	27 (43.55)	13 (30.95)		
Female	475 (61.77)	26 (53.06)	148 (60.41)	237 (63.88)	35 (56.45)	29 (69.05)		
Work content, *n* (%)							H = 3.52	0.47
Physical therapist	476 (61.89)	32 (65.30)	142 (57.96)	231 (62.26)	44 (70.96)	27 (64.28)		
Occupational therapist	87 (11.31)	2 (4.08)	31 (12.65)	43 (11.59)	6 (9.67)	5 (11.90)		
Speech therapist	101 (13.13)	11 (22.44)	34 (13.88)	48 (12.93)	4 (6.45)	4 (9.52)		
Cardiopulmonary therapist	33 (4.29)	0 (0)	14 (5.71)	16 (4.31)	2 (3.22)	1 (2.38)		
Postpartum rehabilitation therapist	9 (1.17)	2 (4.08)	2 (0.81)	3 (0.81)	2 (3.22)	0 (0)		
Pediatric therapist	22 (2.86)	1 (2.04)	8 (3.26)	12 (3.23)	0 (0)	1 (2.38)		
Prosthetic orthotist	12 (1.56)	1 (2.04)	4 (16.00)	6 (1.61)	0 (0)	1 (2.38)		
Concurrent post	29 (3.77)	0 (0)	10 (4.08)	12 (3.23)	4 (6.45)	3 (7.14)		
Kind of work unit, *n* (%)							H = 8.86	0.06
Public hospital	602 (78.28)	42 (85.71)	197 (80.41)	284 (76.55)	52 (83.87)	27 (64.29)		
Private hospital	167 (21.72)	7 (14.29)	48 (19.59)	87 (23.45)	10 (16.13)	15 (35.71)		
Type of work unit, *n* (%)							H = 5.97	0.20
General hospital	591 (76.85)	43 (87.76)	194 (79.18)	275 (74.12)	49 (79.03)	30 (71.43)		
Rehabilitation hospital	127 (16.51)	5 (10.20)	33 (13.47)	70 (18.87)	9 (14.52)	10 (23.81)		
Rehabilitation clinic	43 (5.59)	1 (2.04)	13 (5.31)	24 (6.47)	3 (4.84)	2 (4.76)		
Special hospital	8 (1.04)	0 (0.00)	5 (2.04)	2 (0.54)	1 (1.61)	0 (0.00)		
Workplace location, *n* (%)							H = 5.00	0.28
Capital city	289 (37.58)	24 (48.98)	99 (40.41)	130 (35.04)	22 (35.48)	14 (33.33)		
Non-capital city	480 (62.42)	25 (51.02)	146 (59.59)	241 (64.96)	40 (64.52)	28 (66.67)		
Job title, *n* (%)							H = 29.93	<0.001
Technician	73 (9.49)	6 (12.24)	14 (5.71)	35 (9.43)	9 (14.52)	9 (21.43)		
Technologist	299 (38.88)	8 (16.33)	85 (34.69)	161 (43.40)	28 (45.16)	17 (40.48)		
Technologist-in-charge	317 (41.22)	27 (55.10)	108 (44.08)	143 (38.54)	24 (38.71)	15 (35.71)		
Associate senior technologist	69 (8.97)	5 (10.20)	31 (12.65)	31 (8.36)	1 (1.61)	1 (2.38)		
Senior technologist	11 (1.43)	3 (6.12)	7 (2.86)	1 (0.27)	0 (0.00)	0 (0.00)		
Major, *n* (%)							H = 4.85	0.30
Rehabilitation therapy	517 (67.23)	34 (69.38)	159 (64.89)	248 (66.84)	43 (69.35)	33 (78.57)		
Physical therapy	22 (2.86)	0 (0)	10 (4.08)	9 (2.42)	2 (3.22)	1 (2.38)		
Speech therapy	14 (1.82)	0 (0)	3 (1.22)	8 (2.15)	2 (3.22)	1 (2.38)		
Occupational therapy	17 (2.21)	1 (2.04)	3 (1.22)	9 (2.42)	2 (3.22)	2 (4.76)		
Acupuncture and manipulation	119 (15.47)	6 (12.24)	39 (15.91)	61 (16.44)	8 (12.90)	5 (11.90)		
Clinical medicine speciality	46 (5.98)	4 (8.16)	16 (6.53)	21 (5.66)	5 (8.06)	0 (0)		
Nursing	34 (4.42)	4 (8.16)	15 (6.13)	15 (4.04)	0 (0)	0 (0)		
Degree, *n* (%)							H = 9.35	0.053
Secondary school diploma	9 (1.17)	0 (0.00)	1 (0.41)	6 (1.62)	1 (1.61)	1 (2.38)		
Associate degree	170 (22.11)	10 (20.41)	41 (16.73)	92 (24.80)	14 (22.58)	13 (30.95)		
Bachelor degree	528 (68.66)	36 (73.47)	180 (73.47)	244 (65.77)	42 (67.74)	26 (61.90)		
Master degree	56 (7.28)	2 (4.08)	22 (8.98)	27 (7.28)	4 (6.45)	1 (2.38)		
Doctor degree	6 (0.78)	1 (2.04)	1 (0.41)	2 (0.54)	1 (1.61)	1 (2.38)		
Monthly income level, *n* (%)							H = 114.81	<0.001
<3,000 RMB	86 (11.18)	1 (2.04)	13 (5.31)	37 (9.97)	16 (25.81)	19 (45.24)		
3,000–5,000 RMB	328 (42.65)	12 (24.49)	75 (30.61)	193 (52.02)	32 (51.61)	16 (38.10)		
5,001–7,000 RMB	206 (26.79)	13 (26.53)	79 (32.24)	102 (27.49)	10 (16.13)	2 (4.76)		
7,001–10,000 RMB	94 (12.22)	14 (28.57)	46 (18.78)	28 (7.55)	3 (4.84)	3 (7.14)		
>10,000 RMB	55 (7.15)	9 (18.37)	32 (13.06)	11 (2.96)	1 (1.61)	2 (4.76)		

Job satisfaction is ordered 1 = very satisfied to 5 = very dissatisfied.

Continuous variables are summarized as median (Q1–Q3) and compared using Kruskal–Wallis tests (H statistics).

Percentages within job satisfaction columns use the total *N* = 769 as the denominator unless otherwise specified, the denominator for percentages in parentheses is the corresponding column *n*.

Two-sided *p*-values are reported; *p* < 0.001 is denoted as “<0.001”.

In the proportional-odds ordinal logistic regression model (Table 3), only monthly income level was independently associated with job satisfaction. Each one-category decrease in monthly income was associated with a 51% reduction in the odds of reporting a higher job satisfaction category (OR = 0.49, 95% CI: 0.42–0.57, *p* < 0.001). Age (OR = 1.00, *p* = 0.91), years of experience (OR = 0.98, *p* = 0.31), and job title (OR = 1.03, *p* = 0.83) were not statistically significant. Variance inflation factors (VIF) for age and job title exceeded 10, indicating multicollinearity; sensitivity analyses excluding these variables did not materially affect results. Results were nearly identical when using robust standard errors clustered at the work-unit level. Model fit indicators were AIC = 1,815.77, BIC = 1,852.93, and McFadden pseudo-R^2^ = 0.066.

**Table 3:** Ordinal logistic regression for factors associated with job satisfaction.

Variables	B	S.E.	OR (95% CI)	*P*
Age	−0.002	0.013	1.00 (0.97–1.03)	0.91
Years of work experience	−0.022	0.021	0.98 (0.94–1.02)	0.31
Monthly income level	−0.720	0.081	0.49 (0.43–0.57)	<0.001
job title	0.029	0.132	1.03 (0.80–1.33)	0.83

Moel fit: *N* = 769; AIC = 1,815.77; BIC = 1,852.93; McFadden pseudo-R^2^ = 0.066.

Sensitivity: Results were nearly identical using robust standard errors clustered at the work-unit level.

job satisfaction is ordered 1 = very satisfied to 5 = very dissatisfied.

Income level and job title were modeled as ordinal predictors (higher values indicate higher income/title).

OR = odds ratio for being in a higher satisfaction category (vs. all lower categories).

Job title and monthly income level are ordinal, with higher values indicating higher title/income.

B = regression coefficient; OR = odds ratio (per 1-unit increase); CI = confidence interval.

Model adjusted for all variables listed.

## 4 Discussion

Through a comprehensive statistical analysis of the fundamental demographics of participants, we identified significant changes in the distribution of gender, age, and Kind of work unit in comparison to the pre-pandemic period. These demographic shifts are closely linked to job satisfaction outcomes, as changes in the proportion of different age groups and economic conditions may affect therapists' professional stability, career development opportunities, and overall satisfaction levels.

In terms of the gender distribution among rehabilitation therapists in the northwestern region of China, the representation of female rehabilitation therapists has consistently been approximately 63% (19). The findings of the current study reveal that the proportion of female rehabilitation therapists stands at 61%. This figure reflects a notable increase relative to the national proportion of 52% recorded prior to the pandemic (18), and it exceeds the representation of male rehabilitation therapists. Female rehabilitation therapists continue to comprise the predominant demographic in the field. There is a significant link between the growing percentage of women and the increasing enrollment rates among women in recent years. In many academic studies on rehabilitation therapy students, the number of female students exceeds that of their male counterparts, resulting in a higher overall representation of female professionals in the workforce (20–22). Moreover, there has been a notable reduction in the proportion of rehabilitation therapists aged below 30 years, with the percentage decreasing from a previous national statistic of 68% (18) to 52%. Several factors may be influencing this trend. Firstly, the employment rate experienced a downturn during the epidemic, resulting in limited job opportunities for recent graduates (23, 24). This situation has consequently led to a decline in the representation of younger rehabilitation therapists within the profession. Secondly, the social and economic impacts of the epidemic were adverse (25), prompting healthcare institutions to reduce their workforce, particularly among younger rehabilitation therapists. These changing age structures and reduced opportunities for newcomers may further explain why younger therapists have a comparatively lower job satisfaction level, as they often face economic uncertainty and limited professional advancement paths during their formative career stages. This trend is also reflected in the finding that 80.7% earn less than 7,000 yuan per month. Furthermore, with regard to the Kind of work unit for rehabilitation therapists, The proportion of rehabilitation therapy professionals employed in private hospitals has increased from 14% (26) before the pandemic to 21% thereafter. This shift can be largely attributed to a decrease in income and a rise in employee turnover during the epidemic (27, 28). Prior to the pandemic, public hospitals experienced a higher income per capita compared to their private counterparts (29), which contributed to a significant psychological disparity during the pandemic among personnel in public institutions. This disparity ultimately resulted in a heightened rate of resignations among public hospital staff.

Simultaneously, the current investigation has revealed a marked increase in the percentage of technologist-in-charge job title from 19.1% (18) and 8.3% (26) to 41.2% following the outbreak. Meanwhile, the percentage of bachelor degrees increased from 56.6% (26) before the epidemic to 68.7% afterward, indicating a positive growth trend. This rise in educational qualifications and professional credentials can be attributed to several factors. Primarily, the self-isolation measures and restrictions on movement during the pandemic provided participants with additional time to pursue academic advancement and professional development (30, 31). Furthermore, the proliferation of online courses during this period facilitated easier access to pertinent knowledge for rehabilitation therapists (32, 33). Regarding the types of work for rehabilitation therapists post-pandemic, changes have also occurred in the Shandong (26) and northwest regions of China (19) compared to pre-pandemic times. The findings of this study conducted post-pandemic reveal notable increments in the proportions of Cardiopulmonary therapists (4.3%), Postpartum rehabilitation therapists (1.2%), and Prosthetic orthotists (1.6%). The increase in the number of rehabilitation therapists specializing in cardiopulmonary care can be largely attributed to the profound effects of COVID-19 on the respiratory system (34). Cardiopulmonary rehabilitation therapy has been shown to alleviate respiratory symptoms (35) and facilitate patient recovery (36), resulting in increased demand for such rehabilitation therapists during the pandemic. Furthermore, in response to the evolving needs dictated by policies in China and the professional development of the field (37), there has been a gradual rise in the demand for rehabilitation therapists. This trend necessitates a more nuanced classification of rehabilitation therapists, leading to an uptick in the proportion of specialists in postpartum rehabilitation and orthotics.

In conducting a comparative analysis as detailed in Table 2, we came across significant statistical differences in age (H = 32.90, *P* < 0.001), years of work experience (H = 32.14, *P* < 0.001), job title (H = 29.93, *P* < 0.001), and monthly income level (H = 114.81, *P* < 0.001). Consequently, our subsequent model construction via ordinal logistic regression incorporated these factors in Table 3. To tackle potential confounding variables, we examined four factors and noted that years of work experience, job title, and monthly income level acted as confounding elements. By integrating these variables into our ordinal logistic regression, we minimized their impact and obtained reliable results. Our final findings indicated that monthly income is a robust and independent determinant of job satisfaction among rehabilitation professionals. Practical implications include: (1) implementing transparent career ladders that link competencies to title progression; (2) introducing structured supervisor support (monthly one-on-ones and peer mentoring); (3) reducing administrative burden through workflow redesign and digital tools; (4) piloting targeted income adjustments or non-monetary recognition (continuing education credits, conference support); and (5) establishing quarterly feedback loops to monitor satisfaction at unit level and act on early warning signals. Other factors, such as age, years of experience, and job title, showed no significant associations in the multivariable model. Although significant multicollinearity was detected between age and job title, sensitivity analyses confirmed that our findings were robust to their exclusion. These results highlight the preeminence of income in influencing job satisfaction, regardless of demographic or professional background. Model fit indices indicated a statistically significant but modest overall fit. The implied likelihood-ratio test against the intercept-only model was highly significant, yet the low pseudo-R^2^ suggests that important determinants of job dissatisfaction remain unmeasured. Additionally, potential confounding factors such as institutional policies, regional economic variations, or professional support systems may have diluted the impact of these variables. A broader sample and more robust research design in future studies could help clarify the role of these demographic characteristics.

Despite careful design and robust statistical analysis, this study has several limitations. First, Because recruitment occurred via open professional WeChat groups, a conventional response rate could not be calculated; network participation and self-selection may limit generalizability beyond therapists active in these online communities. Second, potential biases may arise from self-reported data, which may under- or overestimate certain relationships. Third, Although internal consistency cannot be estimated for a single-item measure, we demonstrated psychometric adequacy through content validity procedures and construct validity evidence. The pattern and magnitude of associations with income, tenure, and job title are aligned with established job satisfaction literature. Future work could add test–retest reliability and, if needed, cross-cultural replication and longitudinal cohorts are needed to strengthen external validity and to examine temporal dynamics.

## 5 Conclusions

This study applied strict inclusion criteria and robust statistical methods to assess the status of rehabilitation therapists in Hunan after the epidemic. By controlling for crucial factors such as professional background and demographic characteristics in our analyses, we provide stronger evidence regarding the influence of monthly income on job satisfaction. Our findings highlight the significance of adequate compensation and institutional support in improving the professional well-being of rehabilitation therapists. Future research should incorporate larger, more diverse samples and advanced longitudinal methods to validate these findings and address potential residual confounding.

## Data Availability

The original contributions presented in the study are included in the article/Supplementary Material, further inquiries can be directed to the corresponding author.
