# Supplementary material for: Investigation and analysis of rehabilitation therapists' current situation in Hunan province after the COVID-19 epidemic: a cross-sectional study
Source: Front Rehabil Sci. 2025 Nov 20;6:1614160. doi: 10.3389/fresc.2025.1614160 (PMC12675395; doi:10.3389/fresc.2025.1614160)
Supplement: Supplementary file 1 [file Table1.docx]

1. **Gender**

Male

Female

1. **Your date of birth**
2. **Kind of work unit**

Public hospital

Private hospital

1. **Type of work unit**

General hospital

Rehabilitation hospital

Rehabilitation clinic

Special hospital

Workplace location

1. **Workplace location**
2. **Work content**

Physical therapist

Occupational therapist

Speech therapist

Cardiopulmonary therapist

Postpartum rehabilitation therapist

Pediatric therapist

Prosthetic orthotist

Concurrent post

1. **Years of working experience**
2. **Job title**

Technician

Technologist

Technologist-in-charge

Associate senior technologist

Senior Technologist

1. **Major**

Rehabilitation therapy

Physical therapy

Speech therapy

Occupational therapy

Major of acupuncture and manipulation

Clinical medicine speciality

Nursing

1. **Degree**

Secondary school diploma

Associate degree

Bachelor degree

Master degree

Doctor degree

1. **Monthly salary level**

< 3000 RMB

3000-5000 RMB

5001-7000 RMB

7001-10000 RMB

> 10000 RMB

1. **Job satisfaction**

Very satisfied

Satisfied

Average

Dissatisfied

Very dissatisfied
