# Supplementary material for: Investigation and analysis of rehabilitation therapists' current situation in Hunan province after the COVID-19 epidemic: a cross-sectional study
Source: Front Rehabil Sci. 2025 Nov 20;6:1614160. doi: 10.3389/fresc.2025.1614160 (PMC12675395; doi:10.3389/fresc.2025.1614160)
Supplement: Supplementary file 2 [file Table2.doc]

Table 1. Distribution of Responses to the Single-Item Job Satisfaction Scale

| **Response Category** | **Frequency** | **Percentage (%)** |
| --- | --- | --- |
| Very dissatisfied | 49 | 6.4 |
| Dissatisfied | 245 | 31.8 |
| Neutral | 371 | 48.2 |
| Satisfied | 62 | 8.1 |
| Very satisfied | 42 | 5.5 |

Note: Distribution of responses on the single-item job satisfaction scale (N=769). No pronounced ceiling (5.5%) or floor effect (6.4%) observed, indicating good scale discrimination.

Table 2. Spearman Correlations between Job Satisfaction and Theoretically Relevant Variables

| **Variable** | **Spearman ρ** | **p-value** |
| --- | --- | --- |
| Monthly income level | -0.39 | <0.001 |
| Years of experience | -0.20 | <0.001 |
| Job title | -0.20 | <0.001 |
| Educational degree | -0.09 | 0.010 |

Note: Spearman’s rank correlations of the job satisfaction item with hypothesized construct-related variables (N=769). Negative signs reflect coding polarity and remain consistent with the hypothesized directions after recoding (higher income/seniority correspond to higher satisfaction). Two-sided tests.

Table 3. Known-Groups Validity: Group Differences in Job Satisfaction

| **Grouping Variable** | **Test** | **Statistic** | **p-value** |
| --- | --- | --- | --- |
| Job title | Kruskal–Wallis | 36.60 | <0.001 |
| Educational degree | Kruskal–Wallis | 9.40 | 0.052 |
| Kind of work unit | Mann–Whitney U | 46178.5 | 0.082 |
| Type of work unit | Kruskal–Wallis | 5.51 | 0.138 |

Note: Nonparametric comparisons of job satisfaction across groups. Significant differences were observed for job title (Kruskal–Wallis χ²=36.6, p<0.001), supporting known-groups validity. No statistically significant differences were found by unit ownership or unit type; educational degree showed a borderline difference (p=0.052).
